# Multimodal Monitoring of Hemodynamics in Neonates With Extremely Low Gestational Age: A Randomized Clinical Trial

**DOI:** 10.1001/jamanetworkopen.2025.4101

**Published:** 2025-04-09

**Authors:** Renjini Lalitha, Eyad Bitar, Matthew Hicks, Aimann Surak, Abbas Hyderi, Dawn Pepper, Po Yin Cheung, Kumar Kumaran

**Affiliations:** 1 Division of Neonatal-Perinatal Medicine, Department of Pediatrics, University of Western Ontario, London, Canada; 2 Division of Neonatal-Perinatal Medicine, Department of Pediatrics, University of Alberta, Edmonton, Canada; 3 Division of Neonatology, Department of Pediatrics, Queen’s University, Kingston, Ontario, Canada

## Abstract

**Importance:**

Evaluation and treatment of transitional circulation in neonates with extremely low gestational age (ELGA) varies greatly across centers.

**Objective:**

To determine whether multimodal hemodynamic monitoring in neonates with ELGA during the transitional period (first 72 hours of life) will improve cardiorespiratory-kidney health by decreasing vasoactive-ventilation-renal (VVR) score at 7 days.

**Design, Setting, and Participants:**

This unmasked, 2-arm randomized clinical trial included neonates born at a gestational age of 23^0^ to 28^6^ weeks and admitted to a neonatal unit in Canada. Patients were enrolled from February 15, 2019, to December 31, 2021, with follow-up completed in April 2022.

**Interventions:**

Neonates in the multimodal arm received early targeted neonatal echocardiography at 18 to 24 and 66 to 72 hours of life and cerebral near-infrared spectroscopy (NIRS) for the first 72 hours. A study guideline incorporating clinical-biochemical and cerebral near-infrared spectroscopy data was used for hemodynamic consultation in the multimodal arm. Neonates in the standard arm received hemodynamic assessment using clinical-biochemical data.

**Main Outcome and Measures:**

The primary outcome was VVR score at 7 days. The VVR score incorporates measures of inotrope use, ventilation support, and kidney function to reflect cardiorespiratory-kidney health, with possible scores ranging from 0 to 69.62 at 7 days; higher scores indicate worse cardiorespiratory-kidney health.

**Results:**

Primary analysis included 132 neonates with ELGA (68 in the multimodal arm and 64 in the standard arm) with mean (SD) gestational age of 26.4 (1.5) weeks (75 [56.8%] male). The mean (SD) VVR score at 7 days was 16.5 (15.4) in the multimodal arm and 18.9 (20.2) in the standard arm (*P* = .45). A day 7 peak VVR score greater than 53 (>95th percentile for VVR in the entire cohort) was seen only in the standard arm (7 of 63 [11.1%] vs 0 in the multimodal arm; *P* = .005) and was associated with a composite outcome of death or severe intraventricular hemorrhage (odds ratio [OR], 12.37; 95% CI, 1.92-79.63; *P* = .001) and bronchopulmonary dysplasia (BPD) (6 of 6 [100%] vs 55 of 116 [47.4%]; *P* = .01). Incidence of BPD was lower in the multimodal arm (26 of 63 [41.3%] vs 36 of 61 [59.0%]; *P* = .04). Logistic regression showed that VVR score at 7 days in the top quartile (adjusted OR [AOR], 11.40; 95% CI, 2.04-63.67), late sepsis (AOR, 65.24; 95% CI, 5.70-748.18), and patent ductus arteriosus treatment after 72 hours of life vs early or no treatment needed (AOR, 7.20; 95% CI, 1.60-32.41) were associated with BPD.

**Conclusions and Relevance:**

In this study of neonates with ELGA, multimodal hemodynamic assessment was not associated with decreased VVR scores at 7 days. However, this approach led to lower incidence of VVR associated with severe adverse outcomes and lower BPD incidence, suggesting further investigation is needed.

**Trial Registration:**

ClinicalTrials.gov Identifier: NCT03841929

## Key Points

**Question:**

Does multimodal hemodynamic assessment during the transitional period (first 72 hours of life) improve cardiorespiratory-kidney health at 7 days in neonates with extremely low gestational age (<29 weeks’ gestation)?

**Findings:**

In this randomized clinical trial of 132 neonates with extremely low gestational age, multimodal hemodynamic assessment in the first 72 hours of life compared with standard hemodynamic assessment using mostly clinical-biochemical data did not clearly improve cardiorespiratory-kidney health at 7 days of life, as suggested by the mean vasoactive-ventilation-renal scores (16.5 vs 18.9).

**Meaning:**

Multimodal hemodynamic assessment in the transitional period may have little to no effect on cardiorespiratory-kidney health at 7 days in neonates with extremely low gestational age.

## Teaser

This randomized clinical trial assesses whether multimodal hemodynamic monitoring during the first 72 hours of life improves cardiorespiratory-kidney outcomes in neonates with extremely low gestational age.

## Introduction

Neonatal transition from the intrauterine to extrauterine environment is a complex, multiorgan process. The heart of a neonate with an extremely low gestational age (ELGA) has physiologically less than adequate myocardial function and must navigate the delicate balance between pulmonary and systemic circulation, increasing the risk of intraventricular hemorrhage (IVH) and parenchymal infarcts secondary to an unbalanced transition, unlike a term neonate.^1,2,3,4^ The 3 organ systems that are acutely impacted by such an imbalance are the cardiovascular, respiratory, and kidney systems.

Typically, assessment of the hemodynamic status of neonates is performed using clinical and physiological parameters, including heart rate, blood pressure, supplemental oxygen, capillary refill time, urine output, and laboratory parameters (base deficit, hemoglobin, and lactate). These parameters have multiple pitfalls in providing real-time assessment, with some being nonspecific and observer dependent.^5^ Blood pressure continues to be used as a common physiological marker for needing cardiovascular intervention in neonates. However, increasing evidence suggests that monitoring blood pressure alone is not enough given that a threshold blood pressure that predicts poor outcomes in neonates is still lacking, and antihypotensive therapy itself is associated with increased risk of death or poor neurodevelopmental outcomes.^6,7,8^

Targeted neonatal echocardiography (TNE), when used in combination with clinical findings, may be an invaluable tool for identifying hemodynamic compromise, guiding therapeutic intervention, and monitoring treatment response.^9,10,11,12,13,14,15^ The role of TNE in studying cerebral, kidney, and gut blood flow is largely limited. This information can be supplemented by near-infrared spectroscopy (NIRS), which is a technique used to monitor regional oxygen saturation, including regional cerebral oxygen saturation (rScO_2_), hence a surrogate of organ blood flow.^16^ Cerebral NIRS offers additional information regarding cerebral perfusion, which supplements data provided by echocardiography and other modalities^17,18^ and has been shown to reduce cerebral hypoxic burden in neonates.^19,20^

Hemodynamic assessment in neonates with ELGA without considering data regarding systemic flow, cardiac output, and organ perfusion is incomplete and may result in unnecessary and potentially harmful management strategies.^21^ On the other hand, delay in the management of hemodynamic instabilities can result in end organ injury. Hence, there is a clear need for a systematic evaluation of the hemodynamic state using a combination of TNE, NIRS, and clinical assessment that will result in a better clinical understanding of physiology during the transitional period. We hypothesized that having a detailed hemodynamic understanding using a multimodal assessment would result in improved cardiorespiratory-kidney health in neonates with ELGA at 7 days of life. The vasoactive-ventilation-renal score (VVR) is a comprehensive scoring system that can demonstrate cardiorespiratory-kidney health by incorporating the amount of cardiovascular support using a vasoactive score (VIS), ventilatory support using ventilation index (VI), and kidney performance using change in creatinine levels (ΔCr). Both the VIS and the VVR score have been validated in the neonatal population.^22,23,24^ The objective of this study was to determine whether multimodal hemodynamic monitoring during the transitional period improves cardiorespiratory-kidney outcomes as evidenced by decreasing VVR scores in neonates with ELGA at 7 days of life.

## Methods

### Study Design

This study was a pragmatic, unblinded randomized clinical trial (RCT) conducted at the Philip C. Etches Stollery neonatal intensive care unit (NICU) at Royal Alexandra Hospital (RAH), Edmonton, Alberta, Canada, which is a 69-bed perinatal unit that specializes in the care of neonates with ELGA and has access to a neonatal hemodynamic consultation service that uses TNE. The trial protocol is available in Supplement 1. This pragmatic RCT was designed to evaluate the study question under real world conditions. While maintianing randomization and strcutured protocols, it allows for flexibility based on participant conditions, making findings more generalizable for routine practice. Pragmatic themes included in this RCT were the use of corrected transcutanoeus carbon dioxide (CO_2_) levels when arterial CO_2_ values were unavailable to calculate VI score (details in the Outcomes section) as well as the use of noninvasive blood pressure values when invasive values were unavailable.

The trial was reviewed and approved by the institutional review board of the University of Alberta, Edmonton, Canada. Deferred consent was considered appropriate by the institutional review board based on 2 justifications. First, cerebral NIRS is increasingly adopted as the standard of care across many North American NICUs as part of regional perfusion assessment in various neonatal disease conditions. Second, TNE is a universally accepted modality for bedside neonatal hemodynamic assessment. The study used these ancillary modalities of hemodynamic assessment in a timely fashion. Under this deferred method, written consent was obtained from substitute decision makers within 7 days of life. The investigators and delegates obtained written consent at the earliest opportunity after addressing questions from parents, which happened within 72 hours in this trial. This article follows the Consolidated Standards of Reporting Trials (CONSORT) guideline.

### Participants

Neonates born at a gestational age (GA) of 23^0^ to 28^6^ weeks and admitted to the RAH-NICU were eligible for the RCT. Neonates with major structural heart diseases (except patent ductus arteriosus [PDA], atrial septal defect, and ventricular septal defect), those deemed nonviable, or those with major congenital and/or chromosomal disorders were excluded.

### Randomization

The trial used simple computer-generated randomization to assign eligible neonates with ELGA to the study groups. Sealed opaque envelopes were opened when eligibility was confirmed. Neonates were assigned within 6 hours of life to either receive multimodal hemodynamic assessment (multimodal arm) or be treated according to the standard approach used in the RAH-NICU (standard arm). Multiple births were randomized to the same arm.

### Study Procedure

Neonates with ELGA recruited into the standard arm had continuous cardiorespiratory monitoring using electrocardiography, pulse oximetry, and blood pressure monitoring, either invasively via an arterial line or noninvasively as the standard of care practice for neonates with ELGA at the local NICU. TNE studies were performed only at the discretion of the clinical team. While the assessment of hemodynamic instability included a combination of clinical-biochemical parameters of perfusion, including blood pressure, the decision to start treatment with an inotropic agent was based predominantly on mean blood pressure less than the GA in the first 72 hours of life after volume expansion with normal saline.

For the multimodal arm, in addition to the outlined cardiorespiratory monitoring, neonates with ELGA also had rScO_2_ monitoring using a neonatal NIRS sensor, initiated within 6 hours of life. A commercially available oximeter (INVOS-5100; Medtronic Inc) was used, and the normal range of rScO_2_ in stable preterm neonates was defined as 55% to 85% during the first 72 hours of life.^25,26^ TNE was performed at 18 to 24 and 66 to 72 hours of life as described in the eMethods in Supplement 2. Zubrow’s normative blood pressure chart (eTable in Supplement 2) was used for blood pressure reference for the multimodal arm. A comprehensive study guideline composed of clinical-biochemical-rScO_2_ data was used by the study team to make hemodynamic interpretation in the multimodal arm (eFigure in Supplement 2).

Each pathway in the study guideline was transcribed into a flash card for quick references when generating hemodynamic conclusions (Supplement 1). The study team was available 24 hours per day, 7 days per week, to provide hemodynamic consultation where the findings were carefully integrated within the clinical context to reach a description of the hemodynamic status along with recommendations, which were provided to the clinical team.

### Outcomes

The primary outcome was VVR score in both study arms. VVR scores were calculated every 6 hours (where applicable) for the first 72 hours of life, and then at 7 days of life using the following formula:

VVR = VIS + VI + (ΔCr × 10).

Possible VVR scores range from 0 to 140.40 at baseline, 0 to 78.44 at 72 hours, and 0 to 69.60 at 7 days, with higher scores indicating worse cardiorespiratory-kidney health. VIS was calculated using vasoactive dose as follows:

VIS = Dopamine (μg/kg/min) + Dobutamine (μg/kg/min) + 100 × Epinephrine (μg/kg/min) + 10 × Milrinone (μg/kg/min) + 10 000 × Vasopressin (U/kg/min) + 100 × Norepinephrine (μg/kg/min).

For patients not receiving vasoactive support at the time of VVR measurement, VIS = 0. VI was measured as follows:

VI = (Ventilator RR) × (PIP-PEEP) × Paco_2_/1000.

For patients who were receiving continuous positive airway pressure at the time of VVR measurement, VI = 0. For those receiving high-frequency ventilation, peak inspiratory pressure–positive end-expiratory pressure (PIP-PEEP) was replaced by mean airway pressure, and a ventilator with respiratory rate (RR) of 60 breaths per minute (which is the maximum ventilator rate on conventional mechanical ventilation) was used for the purpose of calculating VI.

For patients receiving noninvasive mechanical ventilation (MV) with set PIP, a calculated PIP (set PIP − 5 cm H_2_O) was used to account for the attenuation of set ventilator pressures reaching the nares.^27^ Where arterial gas levels for Paco_2_ were not available, transcutaneous carbon dioxide (CO_2_), which was corrected by subtracting 5, was used to reflect closely to arterial CO_2_.

For ΔCr (in milligrams per deciliter), baseline serum creatinine level was subtracted from the subsequent serum creatinine level, which was labeled ΔCr. Baseline creatinine level was measured at 48 to 72 hours and repeated at 6 to 7 days of life as per unit policy. For patients in whom repeated serum creatinine level was no greater than the level at baseline, ΔCr = 0. Prespecified secondary outcomes included duration of MV, length of hospitalization, bronchopulmonary dysplasia (BPD), necrotizing enterocolitis (NEC), retinopathy of prematurity, IVH, periventricular leukomalacia, PDA, pulmonary hemorrhage, and in-hospital mortality.

### Data Collection

Data on the following patient demographics were recorded: GA, birth weight, type of delivery, antenatal morbidities, use of antenatal corticosteroids, antenatal magnesium sulfate use, Apgar scores, and sex. Data pertaining to ventilation, hemodynamic parameters, vasoactive requirement, invasive and/or noninvasive CO_2_ monitoring, and blood gases, where available, were recorded at randomization, every 6 hours until 72 hours of life, and then at 7 days of life. Echocardiographic data from TNE studies were collected. Major morbidity, duration of MV, length of hospitalization, and mortality were collected for exploratory analysis.

### Sample Size

We assumed a 25% reduction in VVR score in the multimodal arm with a mean VVR score of 30.0 in the standard arm and 22.5 in the multimodal arm, α of 5% and 80% power. Therefore the estimated sample size was 128 participants, with 64 in each arm.

### Statistical Analysis

Primary analyses included all randomized participants with consent obtained and a primary outcome of VVR score at 7 days. All statistical analyses were performed with the intercooled Stata, version 18.0 (StataCorp LLC). All tests were 2 sided (where applicable), and significance was defined as *P* < .05. Adjustments for multiple comparisons were not made in the secondary analyses, given the exploratory nature. Univariate and bivariate analysis were used to describe the sample. Continuous variables were summarized with means and SDs (with medians and IQRs for nonnormal distributions). Data were assessed for skewness and transformations performed as appropriate. Categorical variables were summarized with frequencies and percentages. Categorical variables were compared using the Fisher exact test and continuous variables using 2-sided *t* test. Nonparametric comparison of medians using the Wilcoxon rank sum test was also use for moderately skewed variables. Exploratory multivariable logistic regression models were developed using forward and backward stepwise methods to examine the associations among survival, BPD, NEC, the use of inotropes, VVR score, and clinical variables. The association between different VVR score cutoffs (>48 representing the 95th percentile for the entire study cohort, and literature-derived^22^ >53 associated with adverse postoperative outcomes in neonates undergoing cardiac procedures) and neonatal outcomes was assessed. Separate models were developed to include explanatory variables in the first 6 hours of life, to 7 days of life, and during the entire hospitalization.

## Results

**Figure 1.**
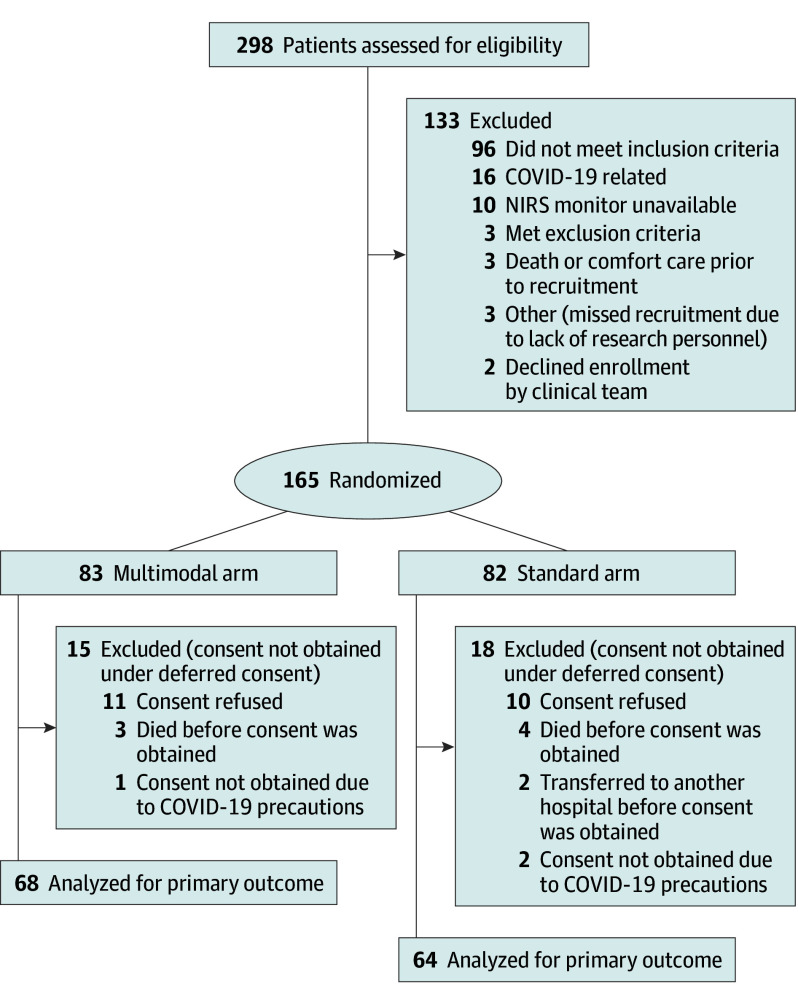
Consort Flow Diagram Of 165 randomized participants, 132 were included in the primary analysis as randomized consented participants. NIRS indicates near-infrared spectroscopy.

A total of 298 neonates born at a GA of 23^0^ to 28^6^ weeks were screened during the study period (February 15, 2019, to December 31, 2021, with the last participant discharged from study site in April 2022). Of 165 neonates with ELGA randomized, 68 in the multimodal arm and 64 in the standard arm were included in the primary analysis as randomized-consented participants (Figure 1). Among the 132 randomized neonates, mean (SD) GA was 26.4 (1.5) weeks; 57 (43.2%) were female and 75 (56.8%) were male.

**Figure 2.**
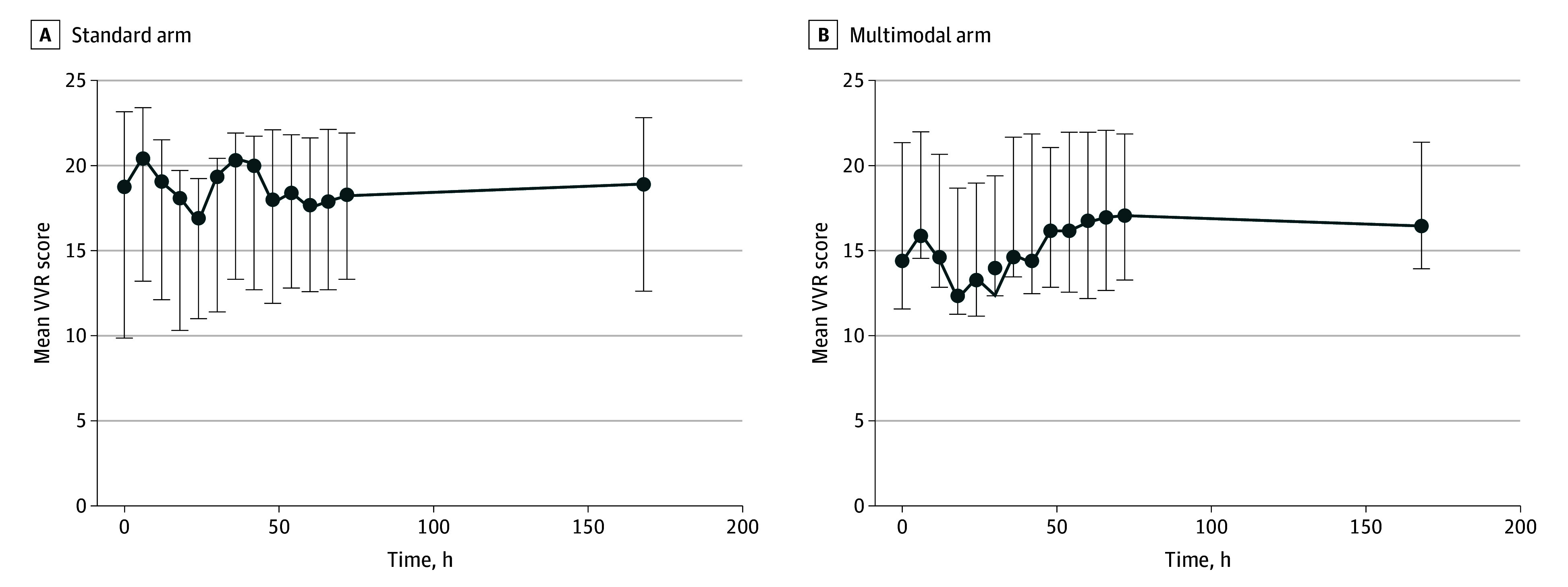
Line Plots Comparing Mean Vasoactive-Ventilation-Renal (VVR) Scores During the First 72 Hours and at Day 7 Possible VVR scores range from 0 to 140.40 at baseline, 0 to 78.44 at 72 hours, and 0 to 69.60 at 7 days, with higher scores indicating worse cardiorespiratory-kidney health. The mean VVR scores were lower in multimodal arm compared with standard arm. Error bars indicate 95% CIs.

Table 1 describes the baseline characteristics of each group. The mean (SD) GA was 26.6 (1.4) weeks in the multimodal arm and 26.3 (1.7) weeks in the standard arm, while mean (SD) birth weight was 947 (233) g in the multimodal arm and 919 (254) g in the standard arm. Notably, 10 neonates (15.6%) in the standard arm had TNE performed at clinical discretion. We examined the longitudinal change in VVR scores across time in both arms (Figure 2) and found comparatively lower mean VVR scores in the multimodal arm across time but without statistical significance.

**Table 1.** Baseline Characteristics of Study Participants at Randomization

Variable	No./total No. (%) of participants	
	Multimodal arm (n = 68)	Standard arm (n = 64)
Gestational age (SD), wk	26.6 (1.4)	26.3 (1.7)
Birth weight (SD), g	947 (233)	919 (254)
Sex		
Male	36/68 (52.9)	39/64 (60.9)
Female	32/68 (47.1)	25/64 (39.1)
Use of antenatal corticosteroids	64/67 (95.5)	60/64 (93.8)
PROM >18 h	20/68 (29.4)	28/64 (43.8)
Suspected or confirmed chorioamnionitis	4/67 (6.0)	10/64 (15.6)
5-min Apgar score <7	25/68 (36.8)	30/64 (46.9)
DCC >30 s	39/55 (70.9)	36/53 (67.9)
SNAPE-II score, mean (SD)^a^	30.0 (18.4)	35.1 (22.1)
Use of supplemental oxygen^b^	62/66 (93.9)	62/64 (96.9)
Noninvasive respiratory support	47/65 (72.3)	37/60 (61.7)
Baseline VVR score, mean (SD)^c^	14.4 (20.9)	18.8 (27.1)

Abbreviations: DCC, delayed cord clamping; PROM, prolonged rupture of membranes; SNAPPE-II, Score for Neonatal Acute Physiology with Perinatal Extension-II; VVR, vasoactive-ventilation-renal.

^a^ Scores range from 0 to 76, with higher scores indicating higher disease severity.

^b^ Fractional inspired oxygen greater than 21%.

^c^ Scores range from 0 to 140.40 at baseline, with higher scores indicating worse cardiorespiratory-kidney health.

### Primary and Secondary Outcomes

All neonates had the primary outcome of the VVR score available at 7 days. Mean VVR scores were comparable between the 2 arms (multimodal, 16.5 [15.4] vs standard, 18.9 [20.2]; *P* = .45). VI and VVR scores remained moderately rightward skewed, although data transformation and nonparametric comparisons of medians were comparable (Table 2). Peak VVR greater than 53, which represented greater than the 95th percentile for VVR score for our entire cohort, was significantly more common in the standard arm (7 of 63 [11.1%]) than in the multimodal arm (0; *P* = .005). While BPD incidence was lower in the multimodal arm vs standard arm (26 of 63 [41.3%] vs 36 of 61 [59.0%]; *P* = .04), other major outcomes were comparable (Table 2).

**Table 2.** Comparison of Neonatal Outcomes Between Multimodal and Standard Approach Arms

Variable	Multimodal arm (n = 68)	Standard arm (n = 64)	*P* value
VI score at 72 h			
Mean (SD)	16.2 (15.7)	17.9 (15.5)	.55
Median (IQR)	13.8 (0-30.8)	19.7 (0.35-28.5)	.61^a^
VI score at 7 d			
Mean (SD)	16.6 (15.0)	17.6 (18.8)	.76
Median (IQR)	14.9 (1.4-27.5)	13.5 (0-33)	.73^a^
VVR score at 7 d			
Mean (SD)	16.5 (15.4)	18.9 (20.2)	.45
Median (IQR)	12.8 (1.3-29)	13.5 (0-34.1)	.83^a^
VVR score >53 at 7 d, No./total No. (%)^b^	0	7/63 (11.1)	.005
Change in VVR over 7 d, mean (SD)	1.9 (20.0)	0.5 (30.5)	.76
Use of inotropes within 7 d, No./total No. (%)	11/68 (16.2)	15/64 (23.4)	.29
Death, No./total No. (%)	6/68 (8.8)	3/64 (4.7)	.34
IVH any grade, No./total No. (%)	12/56 (21.4)	17/59 (28.8)	.36
Severe IVH (grade 3-4), No./total No. (%)	4/56 (7.1)	6/64 (9.4)	.74
Composite death or severe IVH (grade 3-4), No./total No. (%)	6/67 (8.9)	6/64 (9.4)	.82
BPD, No./total No. (%)	26/63 (41.3)	36/61 (59.0)	.04
NEC, No./total No. (%)	4/67 (6.0)	4/64 (6.3)	.94
ROP laser surgery, No./total No. (%)	3/51 (5.9)	8/57 (14.0)	.16
PDA treated, No./total No. (%)	27/68 (39.7)	31/64 (48.4)	.31
Mechanical ventilation, mean (SD), d	31.3 (94.6)	36.2 (73.6)	.73
Length of hospital stay, mean (SD), d	98.8 (103)	107.5 (73.6)	.57

Abbreviations: BPD, bronchopulmonary dysplasia; IVH, intraventricular hemorrhage; NEC, necrotizing enterocolitis; PDA, patent ductus arteriosus; ROP, retinopathy of prematurity; VI, ventilation index; VVR, vasoactive-ventilation-renal.

^a^ Calculated using nonparametric comparison of medians, Wilcoxon rank sum test.

^b^ VVR score greater than 53 at 72 hours was associated with increased mortality and/or length of stay in the intensive care unit among neonates after cardiac surgical procedures.^28^

### Exploratory Analysis

Literature supports the association of a VVR score greater than 53 with adverse postoperative outcomes in neonates undergoing cardiac procedures.^22^ We found higher odds of any grades of IVH (odds ratio [OR], 10.46; 95% CI, 1.14-96.37), composite outcome of death and severe IVH (OR, 12.37; 95% CI, 1.92-79.63), and BPD (6 of 6 [100%] vs 55 of 116 [47.4%] *P* = .01) at this cutoff (Table 3). Additionally, VI represented the greatest component of the VVR score at every time point with minimal contribution from others. In multivariable logistic regression analyses, 3 models for BPD were developed after controlling for sex and birth weight within the first 6 hours of life, within the first 7 days of life, and during total hospitalization. Corrections for multiple comparisons were not made due to the exploratory nature of the analysis. Explanatory variables in the first 6 hours of life that held in the BPD model included randomization to the multimodal arm (adjusted OR [AOR], 0.34; 95% CI, 0.12-0.97), use of antenatal magnesium sulfate (AOR, 0.21; 95% CI, 0.05-0.97), and noninvasive ventilation in the first 6 hours (AOR, 0.19; 95% CI, 0.07-0.55). The BPD model that included variables up to 7 days included randomization to the multimodal arm (AOR, 0.31; 95% CI, 0.10-0.91), VVR score at 7 days in the top quartile (AOR, 8.03; 95% CI, 1.72-37.5), and noninvasive ventilation in the first 6 hours (AOR, 0.24; 95% CI, 0.08-0.71). The final BPD model with variables available throughout the hospital included use of antenatal magnesium sulfate (AOR, 0.11; 95% CI, 0.02-0.69), VVR score at 7 days in the top quartile (AOR, 11.40; 95% CI, 2.04-63.67), sepsis after 3 days of life (AOR, 65.24; 95% CI, 5.70-748.18), and treatment of PDA after 72 hours of life compared with early or no treatment required during hospital course (AOR, 7.20; 95% CI, 1.60-32.41).

**Table 3.** Comparison of VVR Score Greater Than 53 and Neonatal Outcomes^a^

Variable	No./total No. (%) of patients			OR (95% CI)	*P* value
	All	VVR>53	VVR≤53		
Death	9/130 (6.9)	1/7 (14.3)	8/123 (6.5)	2.39 (0.25-22.71)	.43
IVH any grade	49/125 (39.2)	6/7 (85.7)	43/118 (36.4)	10.46 (1.14-96.27)	.009
Severe IVH (grade 3-4)	10/113 (8.8)	3/6 (50.0)	7/107 (6.5)	14.29 (2.14-95.26)	<.001
Death or severe IVH (grade 3-4)	11/113 (9.7)	3/6 (50.0)	8/107 (7.5)	12.37 (1.92-79.63)	.001
BPD	61/122 (50.0)	6/6 (100)	55/116 (47.4)	NA^b^	.01
NEC	8/129 (6.2)	0	8/122 (6.6)	NA^c^	>.99
ROP surgery	11/106 (10.4)	2/6 (33.3)	9/100 (9.0)	5.06 (0.78-32.84)	.05
PDA treated	57/130 (43.8)	5/7 (71.4)	52/123 (42.3)	3.41 (0.62-18.68)	.13

Abbreviations: BPD, bronchopulmonary dysplasia; IVH, intraventricular hemorrhage; NA, not applicable; NEC, necrotizing enterocolitis; PDA, patent ductus arteriosus; ROP, retinopathy of prematurity; VVR, vasoactive-ventilation-renal.

^a^ VVR score greater than 53 at 72 hours was associated with increased mortality and/or length of stay in the intensive care unit among neonates after cardiac surgical procedures.^28^

^b^ No patient without BPD had VVR scores greater than 53.

^c^ No patient with NEC had VVR scores greater than 53.

## Discussion

Our trial evaluated the impact of multimodal hemodynamic assessment in the first 72 hours of life in neonates with ELGA on intermediate and short-term outcomes. Using a combination of rScO_2_ monitoring, longitudinal TNE assessment, and clinical and biochemical parameters that reflect perfusion, we investigated the impact of such comprehensive assessment during the transitional period on neonatal cardiorespiratory-kidney health at 7 days of life and neonatal outcomes. The multimodal hemodynamic assessment did not improve overall cardiorespiratory-kidney health at 7 days, as suggested by the nonsignificant decrease in VVR score; however, incidence of BPD was reduced. Post hoc analysis showed a significantly higher incidence of VVR scores greater than 53 in the standard group, which was associated with an increased composite outcome of death or severe IVH and increased BPD. This is the first study, to our knowledge, to show the effects of multimodal hemodynamic assessment on neonatal outcomes through an RCT.

Assessment of hemodynamic status in neonates is typically performed using a combination of clinical and laboratory parameters that have significant intrinsic limitations. Blood pressure continues to be a common threshold for intervention that, without taking into consideration the status of tissue perfusion or regional blood flow, may lead to unnecessary exposure to vasoactive medications that may be potentially harmful to these extremely vulnerable patients.^8,21^ An increasing number of prospective studies have highlighted the potential merits of TNE.^12,13,14,15^ The role of TNE in studying regional blood flow, including the cerebral, kidney, and gut regions, is largely limited.

Regional blood flow assessment using NIRS is increasingly being adopted in several NICUs for various neonatal conditions. rScO_2_ monitoring using NIRS has been studied through well-structured trials with inconclusive results on its benefits in the preterm population.^29,30^ The SafeBoosC II feasibility trial^29^ used rScO_2_ to show a reduced burden of cerebral hypoxia without significantly decreasing brain injury or death but higher incidence of BPD and retinopathy of prematurity in the intervention group. Similarly, the SafeBoosC III trial was also not associated with a lower risk of death or severe brain injury at a postmenstrual age of 36 weeks.^30^ Despite the lack of significant benefit to neonatal outcomes, rScO_2_ monitoring continues to be widely used in several North American NICUs as the standard of care.

Our RCT incorporated rScO_2_, early TNE, and other traditional modalities of hemodynamic assessment to develop a guided approach for hemodynamic interpretation and recommendations during the transitional period. Using such an assessment, we did not find an improvement in cardiorespiratory-kidney well-being as evidenced by a nonsignificant decrease in VVR score at 7 days. This finding underscores the limited benefits of such an assessment toward the intermediate outcome of cardiorespiratory-kidney performance, suggesting an intricate physiology that may require larger sample size to assess its effects.

However, exploratory analysis showed a lower incidence of BPD within the multimodal arm. Additionally, our BPD models found randomization to the multimodal arm was protective, while having a VVR score in the top quartile and receiving PDA treatment after 72 hours compared with early or no treatment was associated with BPD. Our trial incorporated more comprehensive cardiovascular-cerebral hemodynamic assessments to guide treatment for neonates with ELGA during the transitional period, underscoring the benefits of bundling modalities of hemodynamic assessment rather than using a single modality.

Some of the plausible explanations for the decreased incidence of BPD seen in the multimodal arm include changes in respiratory management based on the study guideline and earlier screening and treatment of hemodynamically significant PDA. Such an improvement in BPD incidence was shown by Giesinger et al,^31^ where early hemodynamic screening and targeted treatment of PDA modulated outcomes of death, severe IVH, severe BPD, and NEC among high-risk extremely preterm neonates.

A VVR score greater than 53 has been associated with increased mortality and/or length of hospital stay in the neonatal postoperative cardiac population.^22,23,28^ We found that a VVR score greater than 53 was also associated with death or severe IVH and BPD, and this cutoff was significantly lower in the multimodal group. Furthermore, there was less use of inotropes in the multimodal arm. Such a decrease could be due to use of the normative blood pressure percentile chart in the multimodal arm with lower targets for intervention vs the GA cutoff for blood pressure target within the standard arm. Additionally, the availability of research team members for hemodynamic consultation 24 hours per day, 7 days per week, provided another layer of assessment leading to avoidance of unnecessary inotropic treatments in the multimodal arm without increasing adverse outcomes.

### Limitations

Our study has some limitations. Other than a single institution’s experience, we did not examine rScO_2_ trends and their association with neonatal outcomes, which may provide an intricate physiological change in this unique population. Furthermore, corrected transcutaneous CO_2_ value was used where invasive CO_2_ levels were unavailable every 6 hours, keeping with the pragmatic theme of this trial. Additionally, the primary outcome was the VVR score at 7 days and not 72 hours, due to the kidney component. Two consecutive creatinine levels are required for kidney score calculation. All participants had baseline creatinine levels measured at 48 to 72 hours of life and repeated on days 6 to 7 as per unit policy. Hence, a complete VVR score incorporating kidney performance was available in only a few participants by 72 hours but for all study participants on day 7. Last, some neonates received TNE, rScO_2_ monitoring, and hemodynamic consultations in the standard arm, introducing contamination. However, we are the first trial, to our knowledge, to examine the effects of multimodal hemodynamic assessment during the transitional period on neonatal outcomes. Our findings reflect a lack of intermediate cardiorespiratory-kidney benefit, but a positive short-term respiratory outcome in a high-risk neonatal population using such an assessment.

## Conclusions

In this RCT involving neonates with ELGA, multimodal hemodynamic monitoring in the transitional period did not improve cardiorespiratory-kidney health at 7 days. These findings support the safety of such comprehensive monitoring in a unique population of neonates with high stakes and its feasibility in clinical practice. While the incidence of BPD was lower in the multimodal arm, larger trials are required to study the effects of such comprehensive hemodynamic monitoring on pertinent neonatal outcomes.

## Notes

Supplement 1.
Trial Protocol

Supplement 2. **eMethods.** Targeted Neonatal Echocardiogram Procedure **eTable.** Normative BP Data **eFigure.** Study Guideline Showing Different Pathways to Guide Study Team With Hemodynamic Interpretation

Supplement 3.
Data Sharing Statement
