# Error in the Visual Abstract

**DOI:** 10.1001/jamanetworkopen.2025.16244

**Published:** 2025-05-15

In the Original Investigation titled “Multimodal Monitoring of Hemodynamics in Neonates With Extremely Low Gestational Age: A Randomized Clinical Trial,”^1^ published April 9, 2025, in the visual abstract, the *P* value for the findings was erroneously displayed as *P* = .045; the correct value is *P* = .45. This article has been corrected.^1^
