# Supplementary material for: Multimodal Monitoring of Hemodynamics in Neonates With Extremely Low Gestational Age: A Randomized Clinical Trial
Source: JAMA Netw Open. 2025 Apr 9;8(4):e254101. doi: 10.1001/jamanetworkopen.2025.4101 (PMC11983231; doi:10.1001/jamanetworkopen.2025.4101)
Supplement: Supplement 1. — Trial Protocol [file jamanetwopen-e254101-s001.pdf]

Multimodal monitoring of hemodynamics in Extremely Low Birth Weight preterm infant in a Canadian tertiary level unit- Non-Blinded RCT (MUSE Trial)

TRIAL REGISTRATION: Clinical Trials.Gov Identifier - NCT03841929

Protocol Date: January 2019

Funding: 1. Women and Children's Health Research Institute,
University of Alberta and Alberta Health Services
2. Neonatal Emerging Research Grant-In-Aid & Special Fund
Division of Neonatal Perinatal Medicine, University of Alberta

Study site: Single Center, Edmonton, Canada

Contents

List of Abbreviations: 4

Background: 5

Rationale: 7

Trial Objectives: 7

- Primary Objective 7
- Secondary Objective 7

Methods: 8

- Study Design 8
- Non-Blinded Randomized Control Trial 8
- Study site 8
- Inclusion Criteria 8
- Exclusion Criteria: 8
- Consent 8
- Study Procedure: 8

Outcome Measures 10

- Primary Outcome 10
- Secondary Outcomes 10

Data Management: 10

- Data Collection and Handling 10

Safety Assessment 11

- Adverse Events (AE) and Serious Adverse Events (SAE) 11
  - **Adverse Events (AE)** 11
  - **Serious Adverse Event (SAE)** 11
  - **Classification of Causality of Adverse Reactions** 12

Statistical Plan and Data Analysis: 13

- Sample Size Calculation for VVR Scores 13
- Analysis 13

Trial Time Frame 13

Study Team 14

References 15

APPENDICES 18

- APPENDIX 1: MUSE Trial Study Flow Chart 18

APPENDIX 2: Site for Application of Cerebral NIRS Probe 19
APPENDIX 3: Targeted Neonatal Echocardiogram procedure 20
APPENDIX 4: Calculation of VVR Scores and VIS Scores 21
APPENDIX 5: Comprehensive Study Guideline for Hemodynamic Assessment 22
APPENDIX 6: Adverse event reporting form 23
APPENDIX 7: Hemodynamic assessment report 24
APPENDIX 8: MUSE Trial Study Guideline-Flash Card 25

List of Abbreviations:

AE: Adverse Events

BPD: Bronchopulmonary Dysplasia

BW: Birth Weight

CRF- Case Report Form

ELGAN: Extremely Low Gestational Age Neonates

GA: Gestational Age

IVH: Intraventricular Hemorrhage

NEC: Necrotizing Enterocolitis

NICU: Neonatal Intensive Care Unit

NIRS: Near Infra-Red Spectroscopy

PDA: Patent Ductus Arteriosus

PVL: Periventricular Leucomalacia

RAH: Royal Alexandra Hospital

RCT: Randomized Controlled Trial

ROP: Retinopathy of Prematurity

$rScO_2$: Regional Cerebral Oxygen Saturation

SAE: Severe Adverse Events

TNE: Targeted Neonatal Echocardiography

VIS: Vasoactive-Inotropic Score

VVR: Vasoactive-Ventilation-Renal

Background:

Neonatal transition from the intra-uterine to extra-uterine environment is a complex, multi-organ process. Of the cardiovascular system, the heart of term neonates is anatomically and functionally mature to make the transition. However, the feto-neonatal transition of extremely low gestational age neonates (ELGAN) may have significant challenges in the context of the physiologically “less than adequate” myocardial function and a delicate balance between pulmonary and systemic blood flow [1]. Further, the brain and cerebrovascular state of ELGAN are particularly vulnerable to the effects of an unbalanced transition. Despite advances in neonatal intensive care that have contributed to a decline in mortality and morbidity, preterm birth remains a major cause of mortality and long-term neurological sequelae [1]. Brain injury in preterm infants is often caused by disturbances in cerebral blood flow and oxygenation [2-4] that happen early in the transition when the majority of intraventricular bleeds and parenchymal infarcts occur. Therefore, it is important to evaluate the systemic and regional hemodynamic state of ELGAN in order to facilitate the neonatal transition.

Neonatal clinicians assess the hemodynamic status of the infant using clinical signs and physiological parameters including heart rate, blood pressure, capillary refill time, urine output and laboratory parameters (base deficit and lactate). While urine output and laboratory parameters do not provide real-time assessment of the hemodynamic status, heart rate and capillary refill time could be non-specific, inaccurate, and observer-dependent, respectively. Blood pressure continues to be used as a common physiological marker for cardiovascular state in neonates. However, there is an increasing amount of evidence that monitoring blood pressure alone is not enough to ensure adequate systemic and cerebral perfusion and oxygenation [5, 6]. Indeed, preterm babies may have “low blood pressure” triggering pharmacological treatment while still maintaining adequate systemic flow, especially cerebral and other organ perfusion as shown by other noninvasive measures like targeted neonatal echocardiography (TNE) and near infrared spectroscopy (NIRS). Interestingly, Baton et al demonstrated that independent of early blood pressure changes, anti-hypotensive therapy exposure in ELGAN was associated with an increased risk of death/neurodevelopmental impairment at 18-22 months corrected age even after controlling for risk factors known to affect survival and neurodevelopment [7].

The use of TNE by neonatal clinicians to evaluate cardiovascular state in neonates is common in many tertiary neonatal intensive care units [8-10]. When used in combination with clinical findings, neonatal echocardiography may be an invaluable tool for the identification of hemodynamic compromise, guiding therapeutic intervention, and monitoring treatment response [9, 10]. There are an increasing number of

prospective studies that highlight the potential merits of TNE in identification of cardiovascular compromise and guiding neonatal cardiovascular care [11-14]. In addition, it has been shown that the use of early echocardiography to provide targeted PDA treatment may result in a reduction of severe intraventricular hemorrhage and pulmonary hemorrhages [12, 13]. The role of TNE to study organ blood flow including cerebral, renal and the gut is largely limited.

NIRS is a technique that can be used to monitor regional cerebral oxygen saturation ($rScO_2$), being both a measure of cerebral oxygenation as well as a surrogate of cerebral blood flow [15]. NIRS monitoring can be applied for prolonged periods of time, even in the most vulnerable infants [16]. It uses multiple wavelengths of NIR light and relies on the distinct absorption spectra of oxygenated and deoxygenated. NIRS offers the ability to assess target organ blood flow. It offers additional information regarding organ perfusion, which supplements data provided by echocardiography and other modalities [17, 18]. In preterm infants, NIRS-derived fractional tissue oxygen extraction and regional cerebral oxygen saturation reference values, particularly over the first 72 hours of life, are emerging [19]. NIRS is being used in many centers including ours, in an ad-hoc manner. In a multicenter, randomized, controlled trial, infants monitored with NIRS and treated for evolving cerebral hypoxia had a lower cerebral hypoxic burden when compared with infants who were not treated based on NIRS findings [20-22]. The long-term benefit of this approach has yet to be elucidated.

There have been attempts to combine the TNE and NIRS studies in preterm neonates to guide management, showing benefits including reduced inotrope use and length of stay. There is a clear need for a systematic evaluation of the hemodynamic state using a combination of TNE, NIRS and clinical assessment that will result in better clinical understanding of the physiology especially during the transitional period by the neonatal clinicians. The assessment of ELGAN hemodynamics without considering data regarding systemic flow, cardiac output and organ perfusion is incomplete and may result in unnecessary and potentially harmful management strategies.

Various scoring measures have been used in neonates and children to objectively categorize the cardiovascular support using inotrope use as a surrogate. Vasoactive inotrope score (VIS) which provides a numerical sum of inotrope doses used, has been used in preterm infants to quantify cardiovascular support in the perioperative period [23]. A more comprehensive score: Vasoactive Ventilation Renal Score (VVR) that incorporates ventilation support and renal function assessment has been used to evaluate children during the perioperative period [24, 25]. There are no neonatal studies using VVR Scores in the transitional period after birth.

The hypothesis is that having a detailed hemodynamic understanding using a multimodal assessment will result in better cardiorespiratory and renal health at 1 week in ELGAN as evidenced by lower VVR Scores.

Rationale:

Using mean BP alone as the indication of treatment of neonatal cardiovascular compromise without taking into consideration the status of tissue perfusion may lead to unnecessary exposure of neonates to vasoactive medication. This medication can be potentially harmful to these extremely vulnerable patients. Analysis of a large neonatal database [26] has demonstrated that treatment of hypotension was associated with an increase in serious brain injury. This held true even after the blood pressure was included in the regression mode, suggesting that it is treatment of hypotension, rather than the presence of hypotension which is harmful. The common interventions, fluid boluses followed by inotropes, could as well be harmful. Observational data has shown an association of fluid boluses with intracranial bleeding and in animal models intraventricular hemorrhage after hypotension can be induced by rapid volume infusion. Fluctuations in blood pressure when inotropes are introduced are well known and could also trigger hemorrhage. Current standard approaches to evaluation and treatment of transitional circulatory problems in the preterm infant may be harmful. Therefore, it is essential that these approaches are adequately investigated.

Trial Objectives:

Primary Objective

To determine whether a multimodal hemodynamic monitoring using combined TNE and cerebral NIRS can reduce inotrope use as evidenced by decreasing VVR Scores in ELGAN within the first one week of life.

Secondary Objective

To validate the use of VVR Scores during the neonatal transition period in ELGAN population.

Methods:

Study Design

Non-Blinded Randomized Control Trial

Study site

This study will be conducted at the Royal Alexandra Hospital Neonatal Intensive Care Unit (RAH NICU) which is a 69-bed unit that specializes in the care of very premature infants. On average, about 150 ELGAN less than 29 weeks are admitted per year to the RAH NICU for specialized neonatal care.

Inclusion Criteria

1. Preterm babies less than 29 weeks ELGAN.
2. Babies born in RAH and admitted to RAH NICU for first 7days of life.

Exclusion Criteria:

1. Structural heart diseases.
2. Major congenital disorder or chromosomal disorder.

Consent

Deferral of consent will be sort owing to the design of the study. Cerebral NIRS is increasingly adopted as standard of care across many North American NICUs and TNE is a universally accepted modality for assessment of hemodynamics which is presently being performed in our NICU. The proposed study is using these existing ancillary modalities of hemodynamic assessment in a timelier fashion. Under deferral of consent, written consent will be obtained within 7 days of delivery, after addressing questions from the parents.

Study Procedure:

This will be a Non-Blind Randomized Control Trial.

The study will involve the following steps (**APPENDIX 1**):

1) The study population will be assigned by simple randomization to either Multimodal arm to receive multimodal hemodynamic assessment using the study guideline (**APPENDIX 5**) or Standard arm to be treated according to the standard approach operative in RAH NICU.

2) ELGANs recruited into Standard arm will have

   a) Continuous cardiorespiratory monitoring using ECG leads or via an Arterial line, and pulse oximetry and intermittent non-invasive blood pressure monitoring as standard of care in the NICU. Continuous arterial blood pressure monitoring will also be obtained as appropriate when an arterial line has been placed under the clinical team's discretion.

   b) TNE studies will be performed at the discretion of clinical team for concerns on cardiorespiratory status of the study population.

   c) The current treatment protocol for hemodynamic compromise at our NICU is starting inotropic support if mean blood pressure (MBP) is less than the gestational age of the infant in the first 72hours of life.

   d) Cerebral NIRS monitoring for $rScO_2$ using neonatal sensors is not routinely used in the NICU and its application for the standard arm will be up to clinical team discretion.

3) ELGANs recruited to the Multimodal arm will have the same monitoring outlined in 2a in a standardized manner.

   a) Continuous cerebral NIRS monitoring for $rScO_2$ using neonatal sensor will be attached to the forehead of the infant for first 72 hours of life (**APPENDIX 2**).

   b) Hemodynamic assessment using TNE (**APPENDIX 3**) will be performed at 18-24 hours and 66-72 hours of life. Additional TNE studies will be performed at the discretion of the clinical team for concerns on cardiorespiratory status of the study population.

   c) Zubrow's Normative BP chart will be used and MBP less than the third centile for the gestational age will be taken as low BP for the study.

   d) Blood pressure values and NIRS data will be integrated along with the clinical/biochemical information every 6 hours as well as whenever there is a deviation from the normal range.

   e) A comprehensive study guideline comprising of the clinical, biochemical and NIRS data will be used by the study team in such cases to derive to the hemodynamic assessment of the study subject.

   f) The findings will be carefully integrated within the clinical context to reach a description of the hemodynamic status of the study infant (**APPENDIX 5 and 7**).

4) All neonates will have standardized assessment for lead related skin abrasions as per standard practice, neonatal pain and stress assessment (NPASS Score) as part of their standard care.

5) Developmental support care will be given by the support person during the TNE assessment as per standard unit practice.

6) The discontinuation of either or both NIRS and TNE will depend on the discretion of the clinical team. The reason for the discontinuation will be sorted. This does not constitute a deviation from the study protocol.
7) The admission, 24 hours, 48 hours, 72 hours and 7days mean and median VVR and VIS scores will be calculated in both Study and Standard arms. (**APPENDIX 4**).
8) Urine output will be monitored during the study period as a surrogate for renal function and urine output less than 1 ml/kg/hr will be considered as oliguria.
9) SNAPPE-II Scores, which is a validated disease severity score in preterm babies, will be calculated in both study and standard arm at admission, 72 hours and 7 days of life.
10) The study team will be available 24/7 for hemodynamic consultation and for the ease of making hemodynamic interpretation, each pathway in the comprehensive guidelines will be transcribed into individual flash cards that will be easily accessible to study team. (**APPENDIX 8**).

Outcome Measures

Primary Outcome

The primary outcome would be VVR Score at 7days in both arms.

Secondary Outcomes

Prespecified secondary outcomes include duration of mechanical ventilation, length of hospital stays, bronchopulmonary dysplasia (BPD), necrotizing enterocolitis (NEC), retinopathy of prematurity (ROP), intraventricular hemorrhage (IVH), periventricular leukomalacia (PVL), patent ductus arteriosus (PDA), pulmonary hemorrhage and mortality in hospital.

Data Management:

Data Collection and Handling

Case report forms will be devised to aid data collection. The following patient demographics will be recorded: gestation, birth weight, type of pregnancy, type of delivery, antenatal morbidities (PIH, placental abruption, chorioamnionitis), antenatal steroids, antenatal $MgSO_4$, Apgar Scores, and gender. Information will be collected about the active clinical issue and disease states at the time of the hemodynamic assessment. Data pertaining to cardiorespiratory support, ventilation requirement, inotropes requirement will be recorded during the first week of life. Data for the TNE cardiac function

studies will be collected in a separate datasheet and continuous NIRS data will be collected for the first 72 hours of life. Major morbidity data like BPD, NEC, ROP, severe brain injury, PDA and mortality will be collected for the purpose of validation of VVR Score in the transitional neonatal period. All data will be entered into REDCap Software and will be securely stored on an institutional computer that is password protected.

Safety Assessment

Adverse Events (AE) and Serious Adverse Events (SAE)

Definitions:

Adverse Events (AE)

Any undesirable event occurring to a participant during a clinical trial, whether or not considered related to the trial intervention.

Serious Adverse Event (SAE)

Any adverse event that results in death; is life-threatening, requires prolongation of existing hospitalization, result in persistent or significant disability or incapacity, or requires intervention to prevent permanent impairment or damage.

Medical and scientific judgement should be exercised in determining whether an event is an important medical event. An important medical event may not be immediately life threatening and/or result in death or hospitalization. However, if it is determined that the event may jeopardize the subject and/or may require intervention, to prevent one of the other serious adverse event outcomes listed below, the important medical event should be reported as serious.

Taking into account the frequently severe initial conditions of infants born between $23^{0/7}$ and $28^{6/7}$ weeks of GA as well as their high risks of complications, the following morbidities or complications have to be routinely reported as AE (APPENDIX 6):

- Important medical events that are not already collected in the neonatal CRFs .
- Any worsening of the medical event, if judged as serious and related, even if already collected in the neonatal CRFs.
- All adverse events that are related or possibly related to cerebral NIRS and TNE assessment.
- The following subset of AEs in the infant: even if already reported in the CRF:
    - Blood in one or both ventricles without dilatation (Grade II) (report as AE).

- Blood in one or both ventricles with dilatation (Grade III) (report as SAE).
- Brain intraparenchymal blood- Grade IV (report as SAE).
- Hydrocephalus (report as SAE).
- Cystic PVL (report as SAE).
- Cerebral Atrophy (report as SAE).
- Intestinal perforation/NEC requiring surgery (report as SAE).
- ROP requiring surgery (report as SAE).
- Pneumothroax requiring chest tube (report as SAE).
- BPD (report as SAE).
- Death (report as SAE).
- Mild skin irritation secondary to Cerebral NIRS probe, intact skin with erythema (report as AE).
- Moderate-Severe skin irritation secondary to Cerebral NIRS probe- peeling skin, denuded skin, exudates, blisters (report as SAE).

Classification of Causality of Adverse Reactions

**Certain**	Event or laboratory test abnormality, with plausible time relationship to the MUSE trial; cannot be explained by disease or other treatments; response to withdrawal plausible (pharmacologically, pathologically); event definitive pharmacologically or phenomenologically (i.e. an objective and specific medical disorder or a recognised pharmacological phenomenon); or rechallenge satisfactory, if necessary.
**Probable / Likely**	Event or laboratory test abnormality, with reasonable time relationship to the MUSE trial; unlikely to be attributed to disease or other treatment; response to withdrawal clinically reasonable; rechallenge not required.
**Possible**	Event or laboratory test abnormality, with reasonable time relationship to the MUSE trial; could also be explained by disease or other treatments; information on drug withdrawal may be lacking or unclear.
**Unlikely**	Event or laboratory test abnormality, with a time to the MUSE trial that makes a relationship improbable (but not impossible); disease or other drugs provide plausible explanations.
**Conditional / Unclassified**	Event or laboratory test abnormality; more data for proper assessment needed; or additional data under examination.
**Not Assessable / Unclassifiable**	Report suggesting an adverse reaction; cannot be judged because information is insufficient or contradictory; data cannot be supplemented or verified.

Statistical Plan and Data Analysis:

Sample Size Calculation for VVR Scores

Assuming 25% reduction in VVR in intervention arm with mean VVR Score of 30 in control arm and 22.5 in intervention arm, sd-15; t test assuming sd1 = sd2 = sd; Ho: m2 = m1 versus Ha: m2 != m1

Study Parameters:

alpha = 0.0500; power = 0.8000; delta = -7.5000; m1 = 30.0000; m2 = 22.5000

sd = 15.0000

Estimated sample sizes: N = 128; N per group = 64

Analysis

All statistical analyses will be performed with the intercooled Stata Version 18.0 (College Station, Texas). All data will be anonymized for analysis and intention to treat analyses will be performed for primary outcome. All the tests will be two-sided (where applicable), and significance will be defined as p-value <0.05. Univariate and bivariate analysis will be done to describe the sample. Categorical variables will be compared by Fisher's Exact Test and continuous variables by Student's t-test (two-sided). Logistic and linear regression models will be developed using forward and backward stepwise methods to examine the association between different VVR cut off (95th centile for the entire study cohort and literature derived cut off of VVR>53[25] that is associated with adverse outcomes in neonatal postoperative cardiac patients) and neonatal outcome. ROC curve will be used for the purpose of validation of the VVR Scores in neonates during the neonatal period.

Trial Time Frame

Trial Stages	Time Frame
Protocol Development	Spring 2018
Ethics Submission	Fall 2018
Trial Recruitment Phase	2019- 2021
Data Cleaning and Analysis	2022-2023
Publication	2023-2024

Study Team

Name	Role/Title	Responsibility
Dr. Renjini Lalitha Kumari, TNE Fellow, Neonatal–Perinatal Medicine	Study Lead	Proposal writing, data analysis, concept, study coordination, technical expertise, manuscript development
Dr. Kumar Kumaran, Clinical Director, Neonatologist, TNE lead, Stollery NICU	Study Lead	Day-to-day leadership, Study Coordination, proposal development, implementation, manuscript development, technical expertise
Dr. Abbas Hyderi Neonatologist, TNE practitioner, Stollery NICU	Co-Investigator	Proposal development, concept, coordinate study, implementation, manuscript development, technical expertise
Dr. Matthew Hicks, Neonatologist and Developmental Pediatrician, Stollery NICU	Co-Investigator	Proposal development, concept, study design and analysis support, manuscript development
Dr. Po-Yin Cheung, Neonatologist and Adjunct Professor, Stollery NICU	Co-Investigator	Proposal development, concept, implementation, manuscript development
Dawn Pepper, Neonatal Nurse Practitioner, Stollery NICU	Co-investigator	Proposal development, concept, implementation, technical expertise, manuscript development

References

1. Van Haastert IC, Groenendaal F, Uiterwaal CS, et al. Decreasing incidence and severity of cerebral palsy in prematurely born children. J Pediatr 2011; 159:86–91. e1
2. Alderliesten T, Lemmers PM, Smarius JJ, van de Vosse RE, Baerts W, van Bel F. Cerebral oxygenation, extraction, and autoregulation in very preterm infants who develop peri-intraventricular hemorrhage. J Pediatr 2013; 162:698–704.e2.
3. Van Bel F, Van de Bor M, Stijnen T, Baan J, Ruys JH. Aetiological role of cerebral blood-flow alterations in development and extension of peri-intraventricular haemorrhage. Dev Med Child Neurol 1987;29: 601–14.
4. Borch K, Lou HC, Greisen G. Cerebral white matter blood flow and arterial blood pressure in preterm infants. Acta Paediatr 2010;99: 1489–92.
5. Alderliesten T, Lemmers PM, van Haastert IC, et al. Hypotension in preterm neonates: low blood pressure alone does not affect neurodevelopmental outcome. J Pediatr 2014; 164:986–91.
6. Logan JW, O'Shea TM, Allred EN, et al.; ELGAN Study Investigators. Early postnatal hypotension is not associated with indicators of white matter damage or cerebral palsy in extremely low gestational age newborns. J Perinatol 2011; 31:524–34.
7. Batton B, Zhu X, Fanaroff J, Kirchner HL, Berlin S, Wilson-Costello D, Walsh M. Blood pressure, anti-hypotensive therapy, and neurodevelopment in extremely preterm infants**.** J Pediatr. 2009 Mar; 154(3):351-7, 357.e1. doi: 10.1016/j.jpeds.2008.09.017. Epub 2008 Nov 20.
8. El-Khuffash AF, McNamara PJ. Neonatologist-performed functional echocardiography in the neonatal intensive care unit. Semin Fetal Neonatal Med 2011;16(1): 50–60
9. Weisz DE, Poon WB, James A, et al. Low cardiac output secondary to a malpositioned umbilical venous catheter: value of targeted neonatal echocardiography. AJP Rep 2014;4(1):23–8.
10. Evans N, Gournay V, Cabanas F, et al. Point-of-care ultrasound in the neonatal intensive care unit: international perspectives. Semin Fetal Neonatal Med 2011; 16(1):61–8.
11. Jain A, Sahni M, El-Khuffash A, et al. Use of targeted neonatal echocardiography to prevent postoperative cardiorespiratory instability after patent ductus arteriosus ligation. J Pediatr 2012;160(4):584–9
12. O'Rourke DJ, El-Khuffash A, Moody C, et al. Patent ductus arteriosus evaluation by serial echocardiography in preterm infants. Acta Paediatr 2008;97(5):574–8

13. Kluckow M, Jeffery M, Gill A, et al. A randomised placebo-controlled trial of early treatment of the patent ductus arteriosus. Arch Dis Child Fetal Neonatal Ed 2014; 99(2): F99–104
14. Carmo KB, Evans N, Paradisis M. Duration of indomethacin treatment of the preterm patent ductus arteriosus as directed by echocardiography. J Pediatr 2009; 155(6):819–22
15. Laura Marie Louise Dix, Frank van Bel, Petra Maria Anna Lemmers. Monitoring Cerebral Oxygenation in Neonates: An Update Front Pediatr. 2017; 5: 46. Published online 2017 Mar 14.
16. van Bel F, Lemmers P, Naulaers G. Monitoring neonatal regional cerebral oxygen saturation in clinical practice: value and pitfalls. Neonatology 2008; 94:237–44.
17. Murkin JM, Arango M. Near-infrared spectroscopy as an index of brain and tissue oxygenation. Br J Anaesth 2009;103(Suppl 1): i3–13.
18. Afif El-Khuffash, Patrick J. McNamara, Hemodynamic Assessment and Monitoring of Premature Infants Clin Perinatol - (2017) -–-http://dx.doi.org/10.1016/j.clp.2017.02.001
19. Alderliesten T, Dix L, Baerts W, et al. Reference values of regional cerebral oxygen saturation during the first 3 days of life in preterm neonates. Pediatr Res 2016;79(1–1):55–64.
20. Ng PC, Lee CH, Lam CW, et al. Transient adrenocortical insufficiency of prematurity and systemic hypotension in very low birthweight infants. Arch Dis Child Fetal Neonatal Ed 2004;89(2): F119–26
21. Pellicer A, Greisen G, Benders M, et al. The SafeBoosC phase II randomised clinical trial: a treatment guideline for targeted near-infrared-derived cerebral tissue oxygenation versus standard treatment in extremely preterm infants. Neonatology 2013;104(3):171–8.
22. Plomgaard AM, van Oeveren W, Petersen TH, et al. The SafeBoosC II randomized trial: treatment guided by near-infrared spectroscopy reduces cerebral hypoxia without changing early biomarkers of brain injury. Pediatr Res 2016;79(4):528–35
23. Jesse Davidson, Suhong Tong, Hayley Hancock, Amanda Hauck, Eduardo da Cruz, Jon Kaufman, Prospective validation of the vasoactive-inotropic score and correlation to short term outcomes in neonates and infants after cardiothoracic surgery Intensive Care Med2012 July; 38(7): 1184–1190. doi:10.1007/s00134-012-2544-x
24. Kyle G. Miletic, MD, Ralph E. Delius, MD, Henry L. Walters, III, MD, and Christopher W. Mastropietro, MD. Prospective Validation of a Novel Vasoactive-Ventilation-Renal Score as a Predictor of Outcomes After Pediatric Cardiac Surgery Ann Thorac Surg 2016;101:1558–63

25. Katherine Cashen.et.al Multicenter Validation of the Vasoactive-Ventilation-Renal score as a predictor of prolonged mechanical ventilation after neonatal cardiac surgery Pediatr Crit Care Med., 2018 Aug 7
26. Wong J, Shah P, Yoon E, Yee W, Lee S, Dow K**.** Inotrope use among extremely preterm infants in Canadian Neonatal Intensive Care Units*.* Am J Perinatol 32, 2015, 32(1): 9-14.
27. LS Owen, C J Morley, P G Davis. Pressure variatin during ventilator generated nasal intermittent positive pressure ventilation in preterm babies. s.l. : BMJ 2010 volume 95;Issue 5, 2010.

APPENDICES

APPENDIX 1: MUSE Trial Study Flow Chart

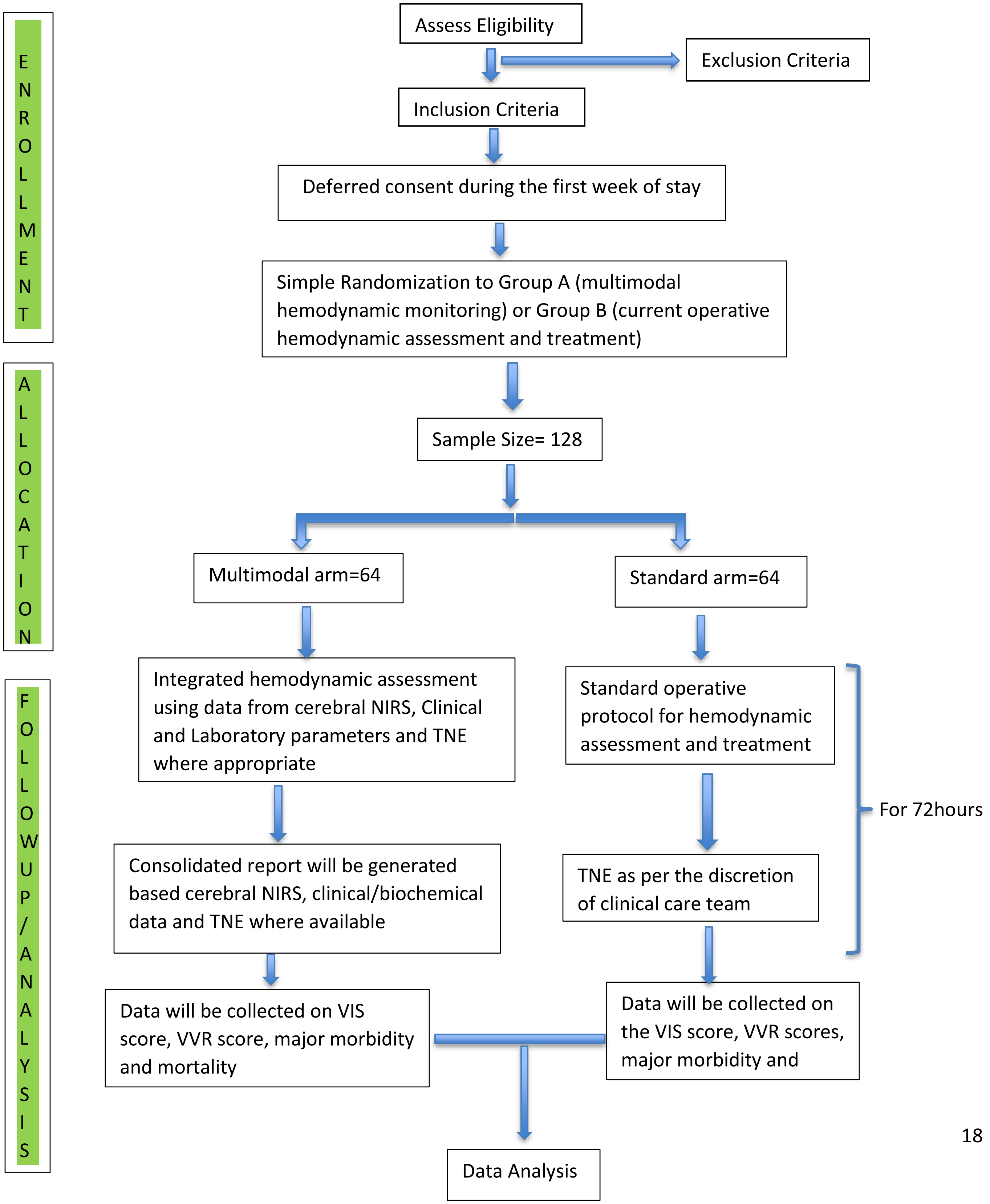

APPENDIX 2: Site for Application of Cerebral NIRS Probe

Cerebral probe will be placed on the midline position for monitoring $rScO_2$ in preterm neonates. Alternative sites could be on the right or left side of the forehead and away from nevi, sinus cavities, the superior sagittal sinus, subdural or epidural hematomas, or other anomalies such as arteriovenous malformations. There are currently no unit specific guidelines for application of neonatal NIRS sensor probe. As such, NIRS probe will be placed similar to a previous trial at our center which is by using Mepitel, one between the skin and NISR probe interface to reduce any skin irritation that could be caused due to the sensor probe. If during the course of the study period center specific guidelines are developed for placement of neonatal NIRS probe that will be adopted into the study, then. Probe sites would be assessed regularly for lead related skin irritation as part of the standard of care practice in the NICU.

The normal reference range of $rScO_2$ for preterm infants varies between 55% and 85% and this would be used for the proposed study. Using NIRS derived $cRSO_2$, Cerebral Fractional Oxygen Extraction (CFOE) will be calculated using the following formula.

$CFOE = (SpO_2 – rScO_2)/ SpO_2$, where

SpO_2= peripheral arterial saturation obtained from pulse oximeter

$rScO_2$= Cerebral oxygen saturation obtained from NIRS monitor

Normal range for CFOE = 0.15- 0.33

APPENDIX 3: Targeted Neonatal Echocardiogram procedure

TNE will be performed using the Vivid E90, GE echocardiography machine. All scans will be performed by either a clinician with extensive training in TNE or by advanced trainees under their supervision. The study will comprise the following standardized assessment:

- Left ventricular performance (Contraction in 4- and 5-chamber views, long and short axis view, VTI, LVO, fractional shortening, IVRT, E:A ratio),
- Right ventricular performance (Contraction 4- and 5-chamber views, long and short axis view with RVO, septum deviation),
- Shunts (PDA: Presence, flow pattern, size and peak velocity MR jet velocity, LA/AO ratio; PFO/ASD/VSD),
- End-organ blood flow: MCA Doppler, Descending Aorta Doppler, SMA, Coeliac axis.

All scans will be reported in real time by attending clinicians dedicated to this service. All studies will be archived on expandable storage of the machines, Xcelera Technology, to store the study images. The scans will all be performed according to unit-specific standardized protocols. Follow-up scans to assess treatment response will be performed according to the clinical situation.

TNE assessment will be performed in 10-15 minute assessment. Babies undergoing TNE assessment are usually contained by facilitated tucking, may be comforted by a parent or nurse and occasionally use of 24% sucrose as needed.

APPENDIX 4: Calculation of VVR Scores and VIS Scores

The VIS is calculated in the following manner:

VIS = dopamine dose (mg/kg/min) + dobutamine dose (mg/kg/min) + 100 x epinephrine dose (mg/kg/min) + 10 x milrinone dose (mg/kg/min) + 10,000 x vasopressin dose (U/kg/min) + 100 x norepinephrine dose (mg/kg/min)

For patients on no vasoactive support at the time of blood gas measurement, VIS equaled zero.

The ventilation index (VI) is measured as follows:

VI = (Ventilator RR) x (PIP - PEEP) x $PaCO_2$ / 1,000

For patients who were extubated to CPAP at the time of blood gas measurement, VI equaled zero.

For those on High Frequency Ventilation, PIP-PEEP will be replaced by Mean Airway Pressure (MAP) and ventilator RR of 60 (which is the maximum ventilator rate on conventional mechanical ventilation) will be used for the purpose of calculating VI score.

For patients on NIMV, a calculated PIP of $5cmH_2O$ less than the ventilator set PIP will be used to account for attenuation of set ventilator pressures reaching the nares [27].

The baseline serum creatinine will be subtracted from serum creatinine (Cr) measurement by 72 hours of life which will be labelled ΔCr. For patients in whom 72 hour serum creatinine measurements were less than or equal to baseline, we will record ΔCr = 0. Creatinine will be measured in mg/dL. In our center creatinine is measured in mmol/L. This will be converted to mg/dL to calculate the score as follows:

serum creatinine (mmol/dL) x 0.0113 = serum creatinine (mg/dL).

Using each of these individual measurements, the VVR Score at the time of each arterial blood gas measurement will be calculated as follows:

VVR = VIS + VI + (ΔCr x 10)

Both VIS and VVR scores have been validated in neonatal population [23, 24, 25].

APPENDIX 5: Comprehensive Study Guideline for Hemodynamic Assessment

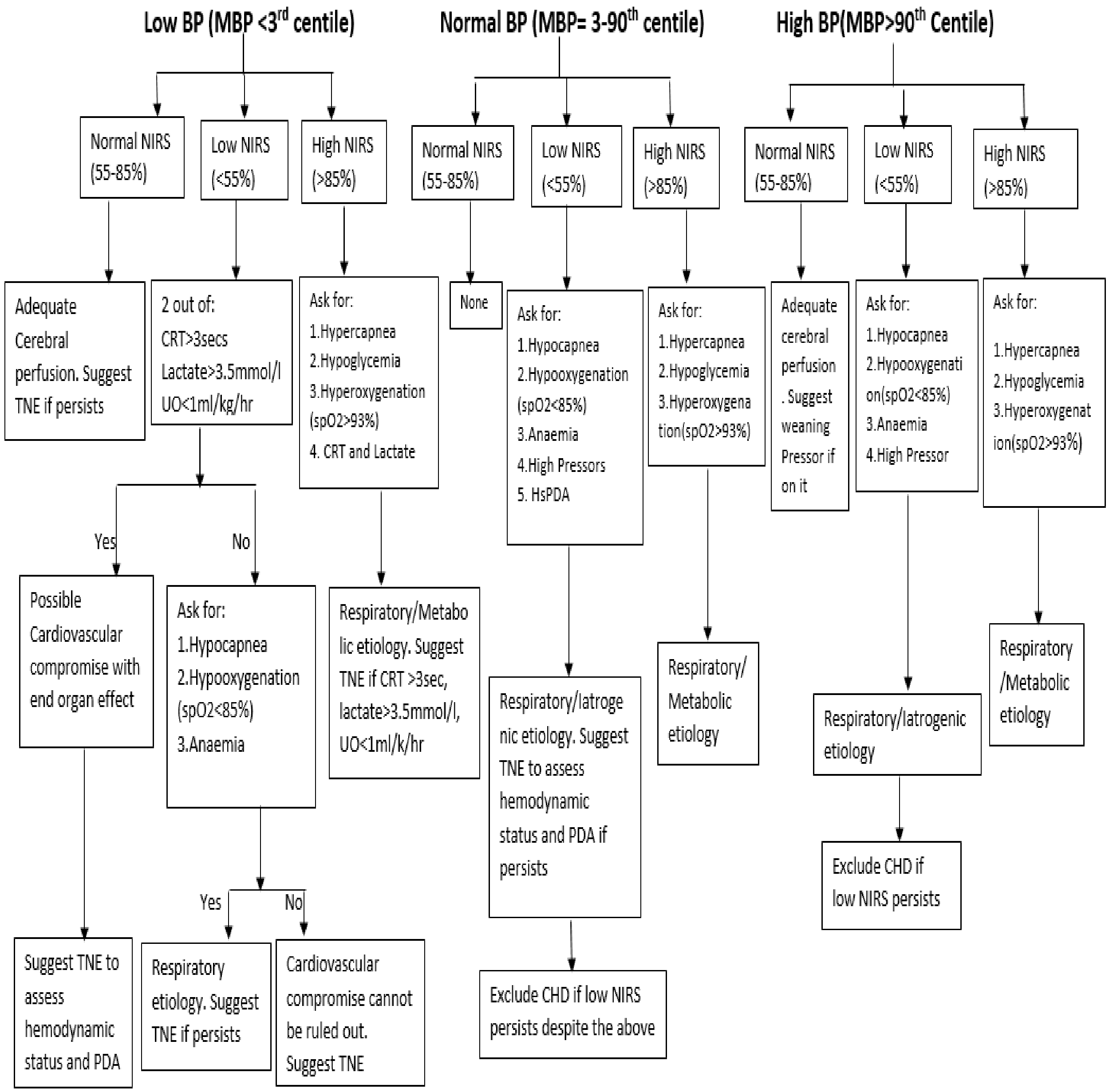

APPENDIX 6: Adverse event reporting form

Adverse Event/Severe Adverse Event Reporting Form

Patient ID: ______________________
DOB: _ _ / _ _ / _ _ _ _

Investigator/name of the person reporting the event: __________________________

Which event is being reported?
Was the event related to the medical device – (NIRS-monitor)?
Yes □
Possibly □
No □
Do not know □

THE EVENT
When did you become aware of the event?
Date and time: _ _ /_ _/_ _ _ _, _ _: _ _

Description of the event:
____
____
____
____
____

Category of the event:
Life-threatening illness or injury □
Prolongation of existing hospitalization □
Permanent impairment of a body structure or body function □
Necessity of medical or surgical intervention to avoid above mentioned □

Was any action taken at the event? Yes □ No □
If yes, describe:
____
____
____
____
____

Event status:
Resolved □
Resolved with sequelae □ describe: ________________________________
Ongoing □
Death □

APPENDIX 7: Hemodynamic assessment report

Hemodynamic Assessment Report:

Date: ___/___/______Time:_____

Clinical/Lab data: GA: ______weeks; B. Wt: _______ gm

BP: _________mmHg; UO: ____ml/k/hr; CRT: ___secs

pCO_2: _____; SpO_2: _____; FiO_2: _____; $rScO_2$: _____

NS bolus: □ Yes, if Yes, no of times: _____ □ No

Blood products: □ Yes, If Yes, specify_______ □ No

Inotropes/Vasopressors: □ Yes □ No

If yes, drug and the dose: _______________________

Lactate: ____mmol/l; Hb: _____ g/l; C/S: ____mmol/l

NIRS: □ Normal □ Low □ High

TNE: □ Yes □ No

TNE Findings: Function________; Cardiac filling_______

LVO______; RVO_______; SVC______; DA flow_______

PV flow________; PDA_________; Pul.pres.__________

Hemodynamic state: □ Adequate SBF □ Low SBF

□ Adequate Brain perfusion □ Low Brain perfusion

□ Other, specify______________________________

Conclusion:

□ Adequate hemodynamic status

□ Compensated CV compromise

□ Decompensated CV compromise

Comments: _______________________________

APPENDIX 8: MUSE Trial Study Guideline-Flash Card

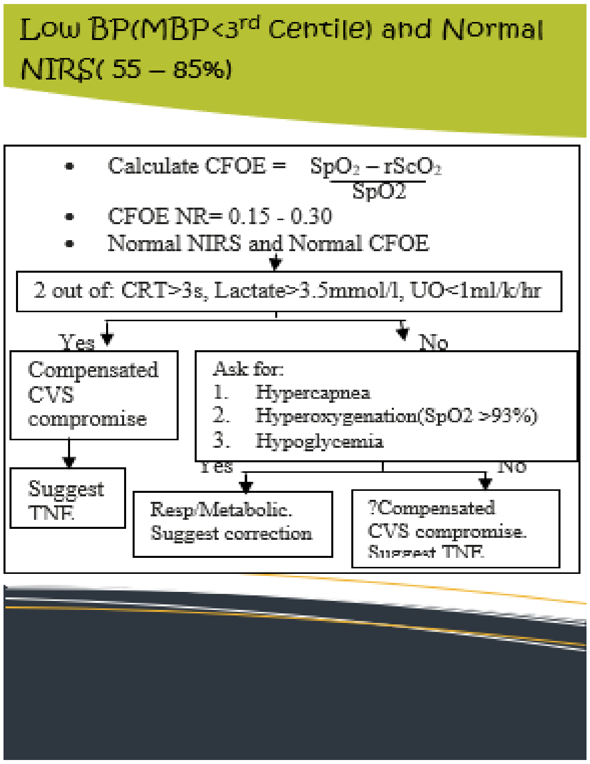

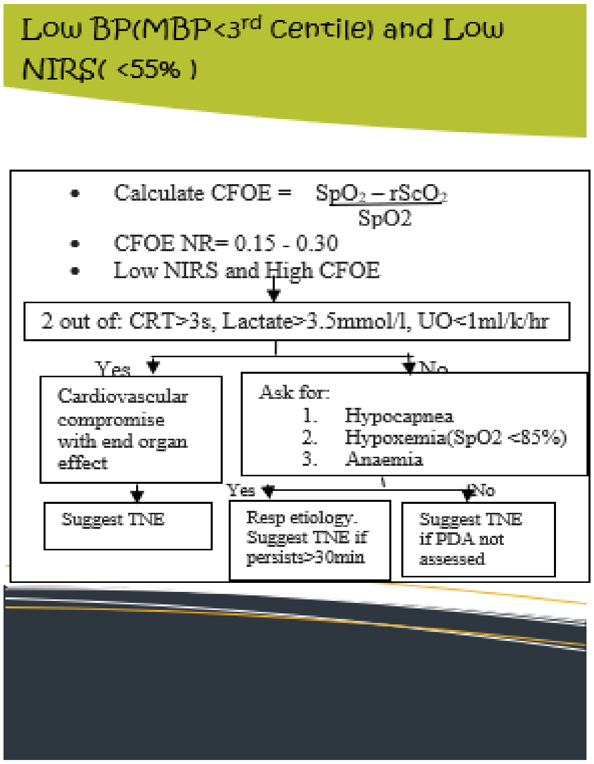

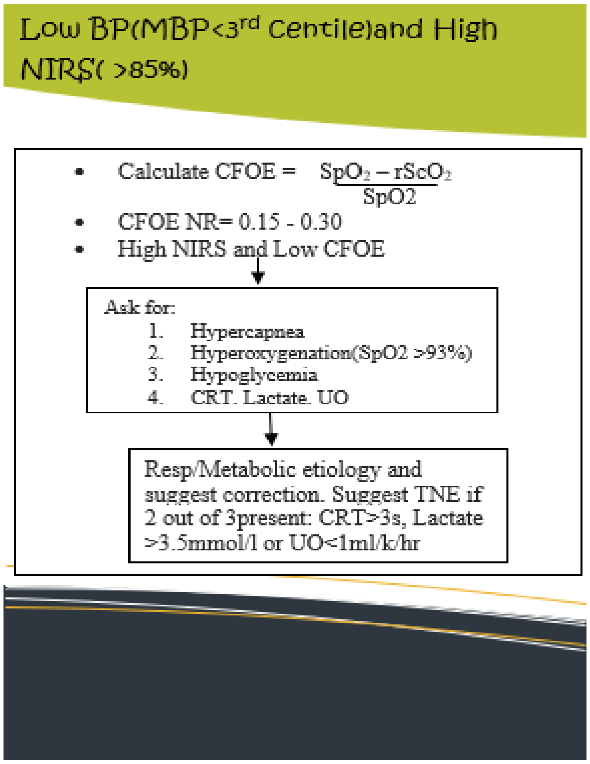

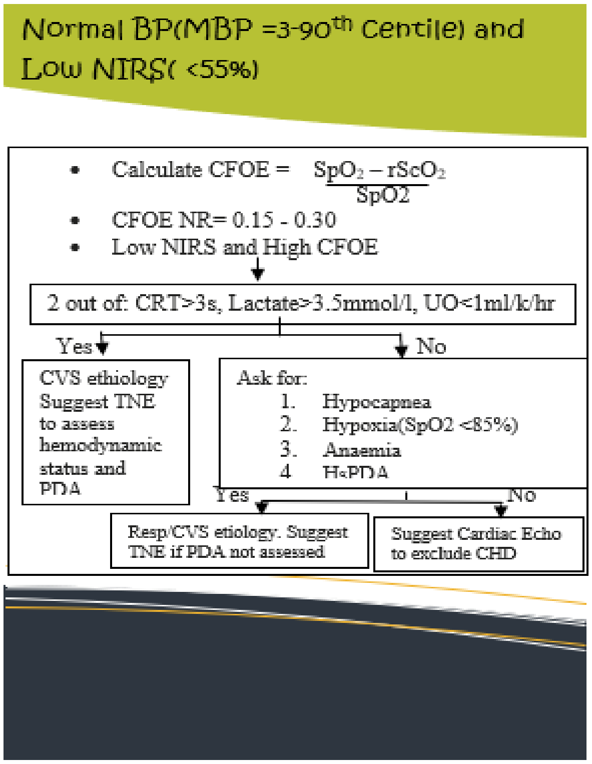

Normal BP(MBP =3-90th centile) and High NIRS(>85%)

- Calculate CFOE = $\frac{SpO_2 - rScO_2}{SpO2}$
- CFOE NR= 0.15 - 0.30
- High NIRS and Low CFOE

↓

Ask for:

5. Hypercapnea
6. Hyperoxygenation(SpO2 >93%)
7. Hypoglycemia

↓

Resp/Metabolic ethiology. Suggest correction

High BP(MBP >90th centile) and Normal NIRS(55 - 85%)

- Calculate CFOE = $\frac{SpO_2 - rScO_2}{SpO2}$
- CFOE NR= 0.15 - 0.30
- Normal NIRS and Normal CFOE

↓

Adequate cerebral perfusion. Suggest weaning pressors if on it

High BP(MBP >90th centile) and Low NIRS(<55%)

- Calculate CFOE = $\frac{SpO_2 - rScO_2}{SpO2}$
- CFOE NR= 0.15 - 0.30
- Low NIRS and High CFOE

↓

Ask for:

1. Hypocapnea
2. Hypoxemia(SpO2 <85%)
3. Anaemia
4. ?High pressors

↓

Resp/ Iatrogenic etiology. Suggest correction and wean pressors if on it

High BP(MBP =3-90th centile) and High NIRS(>85%)

- Calculate CFOE = $\frac{SpO_2 - rScO_2}{SpO2}$
- CFOE NR= 0.15 - 0.30
- High NIRS and Low CFOE

↓

Ask for:

1. Hypercapnea
2. Hyperoxygenation(SpO2 >93%)
3. Hypoglycemia
4. ?High pressors

↓

Resp/Metabolic ethiology. Suggest Correction and wean pressors if on it
