# Supplementary material for: Multimodal Monitoring of Hemodynamics in Neonates With Extremely Low Gestational Age: A Randomized Clinical Trial
Source: JAMA Netw Open. 2025 Apr 9;8(4):e254101. doi: 10.1001/jamanetworkopen.2025.4101 (PMC11983231; doi:10.1001/jamanetworkopen.2025.4101)
Supplement: Supplement 2. — eMethods. Targeted Neonatal Echocardiogram Procedure eTable. Normative BP Data eFigure. Study Guideline Showing Different Pathways to Guide Study Team With Hemodynamic Interpretation [file jamanetwopen-e254101-s002.pdf]

Supplementary Online Content

Lalitha R, Bitar E, Hicks M, et al. Multimodal monitoring of hemodynamics in neonates with extremely low gestational age: a randomized clinical trial. *JAMA Netw Open.* 2025;8(4):e254101. doi:10.1001/jamanetworkopen.2025.4101

eMethods. Targeted Neonatal Echocardiogram Procedure

eTable. Normative BP Data

eFigure. Study Guideline Showing Different Pathways to Guide Study Team With Hemodynamic Interpretation

This supplementary material has been provided by the authors to give readers additional information about their work.

© 2025 Lalitha R et al. *JAMA Network Open*.

eMethods. Targeted Neonatal Echocardiogram Procedure

TNE was performed using the Vivid E90, GE echocardiography machine. All scans were performed by either clinician with extensive training in TNE or by advanced trainees under their supervision. The study comprised of the following standardized assessment:

- Left ventricular performance (Contraction in 4- and 5-chamber views, long and short axis view, VTI, LVO, fractional shortening, IVRT, E:A ratio)
- Right ventricular performance (Contraction 4- and 5-chamber views, long and short axis view with RVO, septum deviation)
- Shunts (PDA: Presence, flow pattern, size and peak velocity MR jet velocity, LA/AO ratio; PFO/ASD/VSD)
- End-organ blood flow: MCA Doppler, Descending Aorta Doppler, SMA, Coeliac axis

All scans were reported in real-time by attending clinicians dedicated to this service. All studies were archived on expandable storage of the machines - Intellispace Cardiovascular Tm 2.3 [Philips Inc., The Netherlands]. technology to store the study images. The scans were performed according to unit-specific standardized protocols. Follow-up scans to assess treatment response were performed according to the clinical situation.

TNE assessments were performed in 10-15 minutes assessment. Babies who underwent TNE assessment were usually contained by facilitated tucking, may be comforted by a parent or nurse and occasionally use of 24% sucrose as needed.

© 2025 Lalitha R et al. *JAMA Network Open*.

eTable. Normative BP Data

Age in weeks	Systolic			Diastolic			Mean(Calculated)		
	Highest	Mean	Lowest	Highest	Mean	Lowes t	highest	mean	lowes t
**24**	68	49	33	46	29	14	53	36	20
**25**	69	51	36	47	30	15	54	37	22
**26**	70	52	38	48	31	17	55	38	24
**27**	71	54	40	49	32	18	56	39	25
**28**	72	55	41	50	33	19	57	40	26
**29**	73	56	42	51	34	20	58	41	27
**30**	75	59	43	52	35	21	60	43	28
**31**	78	61	46	53	36	22	61	44	30
**32**	80	62	48	54	37	23	63	45	31
**33**	81	63	50	55	38	24	64	46	33
**34**	83	66	51	56	39	25	65	48	34
**35**	84	69	52	57	40	26	66	50	35
**36**	87	71	55	58	41	27	68	51	36
**37**	89	72	57	59	42	28	69	52	38
**38**	90	75	59	60	43	29	70	54	39
**39**	91	78	60	60	44	30	70	55	40
**40**	92	80	61	61	44	30	71	56	40
**41**	93	81	62	62	46	31	72	58	41
**42**	95	82	63	63	47	32	74	59	42
**43**	97	83	65	64	48	33	75	60	44
**44**	98	86	67	65	49	34	76	61	45
**45**	100	88	69	66	50	35	77	63	46
**46**	102	89	71	66	51	36	78	64	48

Ref (31)**:** Zubrow AB, Hulman S, Kushner H FB. Determinants of blood pressure in infants admitted to neonatal intensive care units: a prospective multicenter study. Philadelphia Neonatal Blood Pressure Study Group. p. J of perinatology 1995, (6):470-9

© 2025 Lalitha R et al. *JAMA Network Open*.

eFigure. Study Guideline Showing Different Pathways to Guide Study Team With Hemodynamic Interpretation

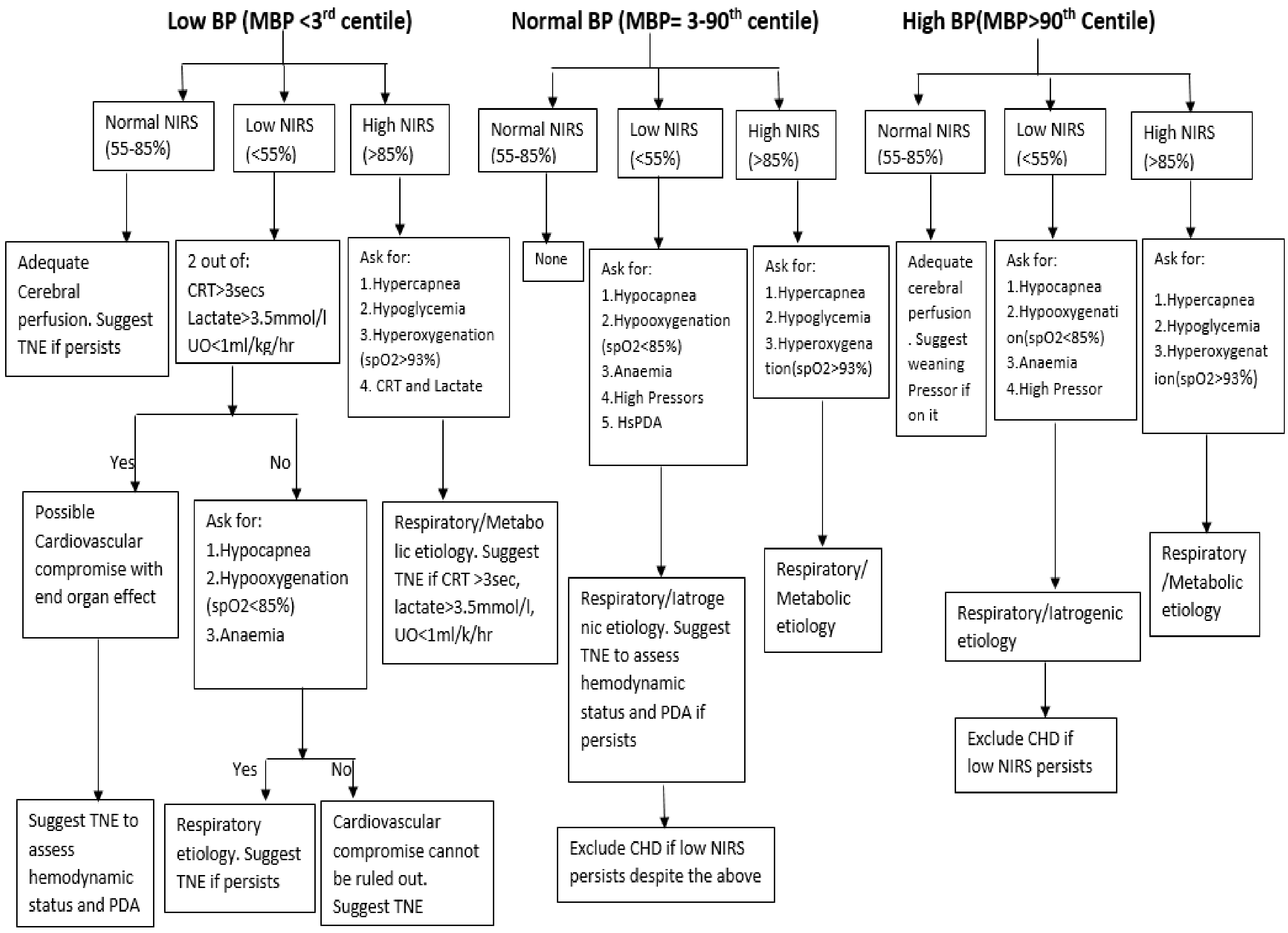

Figure legend: study guideline incorporated cerebral NIRS, MBP and other clinical-laboratory data for a guided approach. Abbreviations: BP-blood pressure, MBP-mean blood pressure, NIRS- Near Infrared Spectroscopy, TNE-Targeted Neonatal Echocardiography, HsPDA-Hemodynamically significant Patent Ductus Arteriosus, PDA- Patent Ductus Arteriosus, CRT-capillary refill time, UO-urine output, spO2-peripheral oxygenation saturation, CHD-congenital heart defect.

© 2025 Lalitha R et al. *JAMA Network Open*.
