# Supplementary material for: Multimodal Monitoring of Hemodynamics in Neonates With Extremely Low Gestational Age: A Randomized Clinical Trial
Source: JAMA Netw Open. 2025 Apr 9;8(4):e254101. doi: 10.1001/jamanetworkopen.2025.4101 (PMC11983231; doi:10.1001/jamanetworkopen.2025.4101)
Supplement: Supplement 3. — Data Sharing Statement [file jamanetwopen-e254101-s003.pdf]

Data Sharing Statement

Lalitha. Multimodal Monitoring of Hemodynamics in Neonates With Extremely Low Gestational Age. *JAMA Netw Open*. Published April 09, 2025. doi:10.1001/jamanetworkopen.2025.4101

Data
Additional Information: ClinicalTrials.gov URL: https://clinicaltrials.gov/ Identifier - NCT03841929
Data available: No

Additional Information
Explanation for why data not available: Alberta Health Services has well defined data sharing regulations that restrict the sharing of individual patient data. Hence, data will not be shared.
